# Five New Species of *Aureoboletus* and *Chalciporus* (Boletaceae, Boletales) and Their Ethnomycological Aspects

**DOI:** 10.3390/jof9101041

**Published:** 2023-10-23

**Authors:** Olivia Ayala-Vásquez, Magdalena Martínez-Reyes, Jesús Pérez-Moreno, César Ramiro Martínez-González, Juan Pablo Pinzón, Javier Isaac de la Fuente, Rigoberto Castro-Rivera, Jesús García-Jiménez, Soledad Balbuena-Carrasco, Eliseete Ramírez-Carbajal, Fuqiang Yu

**Affiliations:** 1 Colegio de Postgraduados, Campus Montecillo, Edafología, Carretera México-Texcoco Km. 36.5, Montecillo, Texcoco CP56230, Mexico; yootspooj@gmail.com (O.A.-V.); martinezreyes2012@gmail.com (M.M.-R.); jdelafuenteitcv@gmail.com (J.I.d.l.F.); lisrc@hotmail.com (E.R.-C.); 2 Departamento de Fitotecnia, Instituto de Horticultura, Universidad Autónoma de Chapingo, Carretera Federal México-Texcoco Km 38.5, Texcoco CP56230, Mexico; ramiro_mg.unam@ciencias.unam.mx (C.R.M.-G.); solbalbuenah@gmail.com (S.B.-C.); 3 Departamento de Botánica, Facultad de Medicina Veterinaria y Zootecnia, Universidad Autónoma de Yucatán, Carretera Mérida-Xmatkuil, Km 15.5, Mérida CP97100, Mexico; juan.pinzone@correo.uady.mx; 4 Instituto Politécnico Nacional, Centro de Investigación en Biotecnología Aplicada, Unidad Tlaxcala, Tepetitla de Lardizábal CP90700, Mexico; rigocastro4@hotmail.com; 5 Tecnológico Nacional de México, Instituto Tecnológico de Ciudad Victoria, Boulevard Emilio Portes Gil #1301Pte, Ciudad Victoria CP87010, Mexico; jgarjim@yahoo.com.mx; 6 The Germplasm Bank of Wild Species, Yunnan Key Laboratory for Fungal Diversity and Green Development, Kunming Institute of Botany, Chinese Academy of Sciences, Kunming 650201, China

**Keywords:** *Aureoboletus*, *Chalciporus*, ectomycorrhizal, ethnomycology, Mexico

## Abstract

Among Boletales, the family Boletaceae has the highest diversity worldwide. Additionally, this fungal group has great ecological relevance because it not only includes mainly ectomycorrhizal but also saprotrophic species. Furthermore, some species are used as food and have sociocultural and economic importance worldwide. In Mexico, the Boletaceae family boasts a substantial number of species, yet our understanding of these species remains far from comprehensive. In this work, by using macro- and micromorphological and phylogenetic analyses of DNA sequences from multi-gene analyses based on ITS, nrLSU, *rpb1*, *rpb2*, and *tef1*, we report five new species belonging to the genera *Aureoboletus* and *Chalciporus: A. ayuukii* and *A. elvirae* from a *Quercus scytophylla* forest, *A. readii* from a mixed forest, *C. perezsilvae* from cloud forest, and *C. piedracanteadensis* from both a mixed coniferous forest and a *Quercus-Pinus* forest. In Mexico, four species of *Aureoboletus* are used as a food source, and in this work, we add another one, *A. readii*, which is traditionally consumed by members of the Tlahuica-Pjiekakjoo culture, who are located in the central part of the country. This work contributes to our knowledge of two genera of Boletaceae in a geographical area that is scarcely studied, and thus, our understanding of its biocultural relevance is enriched.

## 1. Introduction

The family Boletaceae presents the highest diversity within the order Boletales [1], currently having 104 genera and approximately 1345 species [2,3]. Boletales have been extensively studied because of the ecological and economic importance of many species. So far, 83 edible species have been recorded worldwide [4,5]. This group of fungi has great ecological importance because they have established ectomycorrhizal associations with trees of the families Fagaceae, Betulaceae, Pinaceae, Salicaceae, Fabaceae, Nyctaginaceae, and Polygonaceae [1,6]. Currently, six subfamilies are recognized in the Boletaceae family: Austroboletoideae, Boletoideae, Chalciporoideae, Leccinoideae, Xerocomoideae, Zangioideae, and the *Pulveroboletus* group [1].

The *Aureoboletus* Pouzar genus belongs to the subfamily Xerocomoideae, and in the last ten years, 50% of the 35 known species worldwide have been described, with 15 of these species from China [6,7,8]. In North America, 18% of the total known species have been recorded [9,10,11,12]. In Mexico, currently 10 species have been recorded: *Aureoboletus auriflammeus* (Berk. & M.A. Curtis) G. Wu & Zhu L. Yang, *A. betula* (Schwein.) M. Kuo & B. Ortiz, *Aureoboletus garciae* Ayala-Vásquez & Aguirre-Acosta, *A. innixus* (Frost) Halling, A.R. Bessette & Bessette, *A. roxanae* (Frost) Klofac, A. *projectellus* (Murrill) Halling, *A. russellii* (Frost) G. Wu & Zhu L. Yang, *A. singeri* (*Gonz.-Velázq*. & *R. Valenz*.) Har. Takah. & Taneyama, and *A. auriporus* (Peck) Pouzar [12,13,14]. In Mexico, there are records of consumption of *A. auriporus*, *A. russellii*, *A. betula*, and *A. projectellus* [14].

The genus *Chalciporus* Bataille belongs to the subfamily Chalciporoideae, with the type species being *Chalciporus piperatus* (Bull.) Bataille. Currently, 31 species have been described in this genus worldwide [15], 10 of which are distributed in North America and Central America, and only 2 have been recorded in Mexico. This genus is characterized by small to medium basidiomata, with pinkish red to reddish brown pileus, and a pileipellis composed of trichoderm. Its reddish brown, salmon, yellow or chrome hymenophore remains unchanged or stains bluish to dull blue slowly when cut. It is an ecologically important genus because it forms ectomycorrhizal symbioses with conifers, mainly *Pinus* spp. and *Abies* spp. [14,16]. Additionally, mycochemical studies have demonstrated that it contains a few pigments [17], and the edibility of three species have been recorded, mainly in China [4]. This paper, based on macro- and micromorphological, molecular, phylogenetic, and ecological analyses, aims to contribute to the knowledge of the biodiversity and ethnomycology of *Aureoboletus* and *Chalciporus* from Mexico, and presents the dichotomic keys for these genera.

## 2. Materials and Methods

### 2.1. Sampling and Morphological Characterization

Basidiomata were collected from Central and Southern Mexico in the states of Oaxaca state, and Tlaxcala. They were harvested in an oak forest with *Quercus scytophylla*, a montane cloud forest (*Quercus laurina*, *Pinus pseudostrobus*, *Pinus* spp. *Quercus* spp.), and a mixed coniferous forest (*Abies religiosa*, *Quercus laurina*, *Pinus pseudostrobus*) during the rainy season between June and October in 2021–2022. The protocols for sampling macrofungi followed those of Lodge et al. [18]. The color descriptions were according to Kornerup and Wanscher [19]. The microscopical (Leica DM750, Wetzlar, Germany) features of the tubes, pileus, and stipes of dried basidiomata were measured using 5% KOH, Melzer’s reagent, and Congo red. Basidiospore measurements are described as follows: Q = length/width ratio of the basidiospores, with L = average length; W = average width; and N = total basidiospores measured. At least 30 cystidia, basidia, and basidiospores were measured. All specimens were deposited at the mycological herbarium of the National Herbarium of the Instituto of Biología, Universidad Nacional Autónoma de México (MEXU-HO, UNAM), Ciudad of Mexico.

### 2.2. DNA Extraction, Amplification, Sequencing, and Phylogenetic Analysis

DNA extraction, PCR amplification, and genomic DNA sequencing were performed via CTAB [20] using 2–3 mg of dry tissue. DNA quantification was performed using Nanodrop (Thermo, Madison, Wisconsin, EE.UU.). The Internal Transcribed Spacers rDNA-ITS1, 5.8, and rDNA-ITS2 (ITS); the large subunit nuclear ribosomal DNA (nLSU); the largest (*rpb1*) and second largest (*rpb2*) genes for RNA polymerase II; and the translation elongation factor 1-α (*tef1*) were used. The PCR reaction contained the following: enzyme buffer 1×, Taq DNA polymerase, 0.8 mM deoxynucleoside triphosphates (0.2 mM each), 100 ng of DNA, 20 pmol of each primer, and 2 units of GoTaq DNA (Promega, Madison, Wisconsin, USA), for a final volume of 15 µL. The PCR products were verified using agarose gel electrophoresis that was run for 1 h at 95 V cm^−3^ in 1.5% agarose and 1 × TAE buffer (Tris Acetate-EDTA). The products were then dyed with GelRed (Biotium, Fremont, CA, USA) and viewed using a transilluminator (Infinity 3000 Vilber, Loumat, Germany). Finally, the products were purified using the ExoSap Kit (Affymetrix, MA, USA) according to the manufacturer’s instructions and were prepared for the sequencing reaction using the BigDye Terminator Cycle Sequencing Kit v. 3.1 (Applied BioSystems, Woburn, MA, USA). Sequencing was carried out in a genetic analyzer (Sanger sequencing) manufactured by Macrogen Inc. (Seoul, Korea). The sequences were analyzed, edited, and assembled using BioEdit v. 1.0.5 [21] to create consensus sequences. The consensus sequences were compared with those in the GenBank database of the National Center for Biotechnology Information (NCBI) using the BLASTN 2.2.19 tool [22].

To explore the phylogenetic relationships of the new species of *Aureoboletus* (Table 1), an alignment was made based on Farid et al. [11]. Each gene region was independently aligned using the online version of MAFFT v. 7 [23,24,25]. Alignment was reviewed using BioEdit 7.2.5 [21], followed by minor manual adjustments to ensure character homology between taxa. The matrix for nrLSU was formed by 72 taxa (866 characters); for rpb1, it was formed by 48 taxa (687 characters); for rpb2, it was formed by 45 taxa (690 characters); and for tef1, it was formed by 38 taxa (602 characters). The aligned matrices were concatenated into a single matrix (73 taxa, 2845 characters), treating each marker as an independent partition.

To explore the phylogenetic relationships of the new species of *Chalciporus*, an alignment was made based on Zhang et al. [16], (Table 2). Each gene region was independently aligned using the online version of MAFFT v. 7 [23,24,25]. Alignment was reviewed using PhyDE v.10.0 [26], followed by minor manual adjustments to ensure character homology between taxa. The matrix was formed by 13 taxa for ITS (690 characters); for nrLSU, it was formed by 23 taxa (805 characters); for *rpb2*, it was formed by 13 taxa (765 characters); and for *tef*, it was formed by 18 taxa (579 characters). The aligned matrices were concatenated into a single matrix (24 taxa, 2839 characters). Eight partitioning schemes were established: one for ITS, one for nrLSU, three for *rpb2*, and three for *tef1* gene region, which were established using the option to minimize the stop codon with Mesquite v3.70 [27].

The data were analyzed using maximum likelihood (ML) and Bayesian inference (BI). Bootstrap values were estimated using 1000 bootstrap replicates under the heuristic search mode, each with 100 random starting replicates. Maximum likelihood analyses were carried out using RAxML v. 8.2.10 [28] with a GTR + G model of nucleotide substitution. To assess branch support, 10,000 nonparametric rapid bootstrap replicates were run with the GTRGAMMA model, treating each marker as an independent partition, through the platform CIPRES Science Gateway 3.3 [29]. Bayesian inference was carried out using MrBayes v. 3.2.6 ×64 [30] with four chains, and the best evolutionary model for alignment was sought using PartitionFinder v.2.0 [31,32]. The information block for the matrix included two simultaneous runs and four Monte Carlo chains, the temperature was set to 0.2, and sampling was run for 10 million generations (standard deviation ≤ 0.1) with trees sampled every 1000 generations. The first 25% of the samples were discarded as burn-ins, and convergence was evaluated by examining the standard deviation of split frequencies among the runs and by plotting the log-likelihood values from each run using Tracer v. 1.6 [33]. The remaining trees were used to calculate the tree topology based on a 50% majority-rule consensus and posterior probabilities (PP). The trees were visualized and optimized using FigTree v. 1.4.4 [33], and edited using Adobe Illustrator vCS4 (Adobe Systems, Inc., San Jose, CA, USA) or Adobe Photoshop Elements 10 (Adobe Systems, Inc., San Jose, CA, USA). The alignments and phylograms of *Aureoboletus* and *Chalciporus* were uploaded to https://treebase.org/treebase-web/home.html, http://purl.org/phylo/treebase/phylows/study/TB2:S30668, (accessed on 10 August 2023).

## 3. Results

### Phylogenetic Analyses

**Figure 1:**
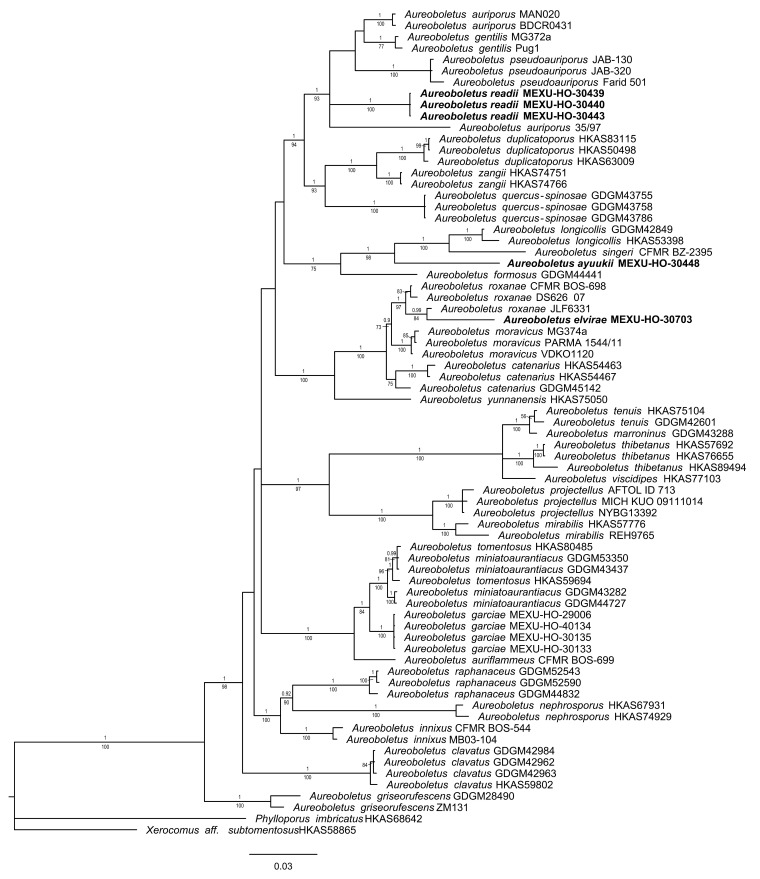
Bayesian analyses of phylogeny based on the concatenated nrLSU, *rpb1*, *rpb2*, and *tef1* sequence alignments. Maximum likelihood and Bayesian analyses recovered identical topologies with respect to the relationships among the main clades of *Aureoboletus*. For each node, the following values are provided: maximum likelihood bootstrap (70%)/Bayesian inference (1 *p*-value). The scale bar represents the expected number of nucleotide substitutions per site. Sequences in bold were generated in this study.

The two phylogenetic analyses based on BI and ML of the dataset recovered similar topologies in *Aureoboletus* (Figure 1). No significant conflict (bootstrap value > 80% vs. PP ≥ 0.9) was detected among the topologies obtained via these separate phylogenetic analyses. The majority-rule consensus tree obtained using Bayesian inference analysis is presented. The two phylogenetic analyses based on ML and BI of the dataset also recovered similar topologies in *Chalciporus*. No significant conflict (bootstrap value > 80%) was detected among the topologies obtained via these separate phylogenetic analyses. The parsimony analysis of the alignments found 856 trees with 212 steps (CI = 0.4852, HI = 0.1011, RI = 0.2024, RC = 0.2810). The best RAxML tree with a final likelihood value of –20,158.025410 is presented. The matrix had 820 distinct alignment patterns, with 2.21% undetermined characters or gaps. The estimated base frequencies were as follows: A = 0.101520, C = 0.207820, G = 0.104302, and T = 0.127041; the substitution rates were as follows: AC = 1.080341, AG = 1.010795, AT = 1.004055, CG = 1.006270, CT = 3.004201, GT = 1.100000; and the gamma distribution shape parameter was α= 0.001020. In the Bayesian analysis, the standard deviation between the chains stabilized at 0.001 after 3.8 million generations. No significant changes in tree topology trace or cumulative split frequencies of the selected nodes were observed after about 0.25 million generations, which were discarded as 25% burn-ins.

*Aureoboletus* Pouzar

Description: This species has small to medium basidiomata with a pileate-stipitate morphology, and its hymenophore is tubular. The pileus is convex, flat-convex, or applanate; its surface is usually glabrous and viscid when wet, tomentose when dry, and finely scaly-squamulose, and the context ranges from whitish, yellowish, golden yellow, to salmon in color, without color change when injured. The hymenophore is adnate to sinuate; its surface’s color ranges from bright yellow, yellow, salmon, to yellow-olivaceous, without color change when injured; the pores are angular to nearly round; and the tubes are concolorous with pores, without change in color when injured. The stipe is central, without change in color when injured, and the basal mycelium is usually white. The basidiospores are usually smooth and rarely longitudinally striate, superficially and discontinuously reticulate, or subfusiform, and the color ranges from light yellow to yellow. The pleurocystidia and cheilocystidia are fusiform-ventricose or clavate, sometimes with long-broad beak, hyaline, and yellowish to yellow in KOH. The pileipellis is usually an ixotrichodermium or trichodermium [1,34]. The clamp connections are absent. The type species is *Aureoboletus gentilis* (Quél.) Pouzar.

Key to the species of *Aureoboletus* in Mexico
1a. Smooth basidiospores ……………………………………………………………………………………………………………………………………………………………………………. 41b. Ornamented basidiospores………………………………………………………………………………………………………………………………………………………………………. 22a. Alveolate to reticulate, ellipsoid, subfusiform basidiospores ………………………………………………………………………………………………………………………………. 32b. Ellipsoid, longitudinally winged basidiospores with pale brown or pale yellow color, and smooth, glutinous, fibrillose stipe………………………………………….. *A. singeri*3a. Reddish, alveolate-to-reticulate stipe that is longitudinally striate, with complete, short, and thick and conspicuous, ellipsoid, cylindrical basidiospores ……….. *A. russellii*3b. Whitish, yellow to orange alveolate to reticulate stipe; basidiospores are superficially and discontinuously reticulate, and ellipsoid to fusiform in shape …………. *A. betula*4a. Yellow, orange-yellow, brown-orange, pale brown, cinnamon, red, reddish-brown, and red-cinnamon basidiomata ……………………………………………………………… 64b. Basidiomata with other colors …………………………………………………………………………………………………………………………………………………………………... 55a. Salmon to peach-colored pileus; salmon hymenophore; pores are salmon, circular, angular to irregular at the edges; concolorous to the hymenophore stipe, with mame-lonate to umbonate pileus, small triangular scales, and crenulate margin …………………………………………………………………………………………………………… *A. elvirae*5b. Vivid blue, violet blue, to light blue pileus; bright yellow hymenophore, and the context is whitish bruising pink to pale red when young, and yellow when mature; 9– 14 × 4–5(–6) µm basidiospores ………………………………………………………………………………………………………………………………………………………………… *A. garciae*6a. Brown-orange, pale brown, cinnamon, red, reddish-brown, red-cinnamon pileus ……………………………………………………………………………………………………… 76b. Vivid yellow, orange-yellow, or orange-brown pileus; golden hymenophore even in dried specimens; and 9–10 (13) × 3– 4 (5) µm, smooth, ellipsoid to subfusiform basidiospores ………………………………………………………………………………………………………………………………………………………………………… *A. auriflammeus*7a. Surface of pileus is viscid, innately fibrillose to reticulate …………………………………………………………………………………………………………………………………... 87b. Surface of pileus is dry, tomentose to areolate at maturity ………………………………………………………………………………………………………………………………….. 98a. Surface of stipe is reticulate at apex or costate ………………………………………………………………………………………………………………………………………………. 108b. Surface of stipe ranges from very viscid to furfuraceous to smooth, is white without color change upon cutting the context, and has (10–)11–13 (–14) × (3.5–) 4–5µm basidiospores ………………………………………………………………………………………………………………………………………………………………………………….. *A. readii*9a. With 12–15(–18) × 5–6 µm basidiospores, the surface of hymenophore is reticulate at apex from the tube remnants, and changes from white bruising brown to brown-orange upon cutting the context ……………………………………………………………………………………………………………………………………………………….. *A. flaviporus*9b. With basidiospores that are 11–16 × 4–6 µm, stipe is costate or reticulate from decurrent tubes, with yellow floccose, and is pale pinkish-brown downward; the hymenophore has a golden-yellow color when dry; the context is yellowish; and the stipe base is pinkish vinaceous or orange-brownish …………………………… *A. auriporus*10a. Basidiomata are medium-sized (>25 mm in diam.), and the surface of the pileus is smooth, tomentose, and dry ………………………………………………………………... 1110b. Basidiomata are small-sized (25 mm in diam.); the surface of the pileus is ornamented, micro-scale to areolate; the hymenophore is vivid yellow; and the basidiospores are amyloid …………………………………………………………………………………………………………………………………………………………………………………. *A. ayuukii*11a. Basidiospores reaching 26 µm in length, thick-walled, with free margin, and pro-jecting and hanging straight down, with dry, smooth, subtomentose, and not-cracked surface …………………………………………………………………………………………………………………………………………………………………………………… *A. projectellus*11b. Basidiospores reaching 17 µm and thin-walle ……………………………………………………………………………………………………………………………………………… 1212a. Distributed in mixed forests, the surface of the pileus is smooth and slightly areo-late at maturity; the color ranges from brown, red to red-brown, rarely with greenish tinges, or white to pale yellow; the pileipellis context is vinaceous; yellow basal mycelium, 9–12.5 × 3–5µm basidiospores ……………………………………………….. *A. innixus*12b. Distributed in cloud forest, the surface of the pileus is granulose and roughened to nearly glabrous at maturity, and its color ranges from yellow-brown, brown-red, to dull orange; the hymenophore is whitish to pale yellow; the stipe base is bulbose; and basidiospores are 9.5–11(–13) × 3.5–4(–4.5) µm ………………………………….. *A. roxanae*

**Figure 2:**
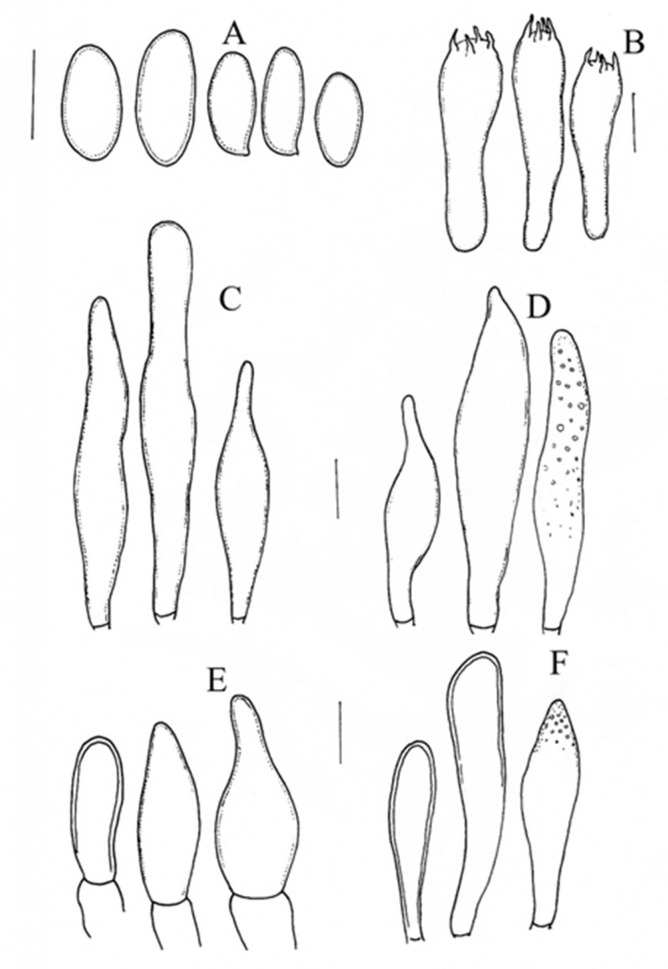
*Aureoboletus ayuukii* (MEXU-HO HO-30448 holotype): (**A**) basidiospores, (**B**) basidia. (**C**) pleurocstidia, (**D**) cheilocystidia, (**E**) pileipellis, and (**F**) caulocystidia. Scale bars: 10 µm.

***Aureoboletus ayuukii*** Ayala-Vásquez, García-Jiménez, de la Fuente, sp. nov. (Figure 2)

MycoBank number: MB 850,013.

Etymology: The name is in honor of the Mixe culture (*Ayuuk*), where this species is currently distributed

Holotype: MEXICO, Oaxaca, Santiago Zacatepec District, Mixistlán de la Reforma Municipality, Santa María Mixistlán Town, Txaajuupa’am Place, 17°08’38” N, 96°06’22” W, alt. 2043 m, 21 November 2016, O. Ayala-Vásquez 797 (MEXU-HO-30448, holotype).

Diagnosis: This species has small yellow basidiomata. The pileus’ surface has furfuraceous to very fine scales; the basidiospores are (14–)15–16–17(–19) × 6.0–7.5 µm; the amyloid is ellipsoid to fusoid; and the pleurocystidia have a very pronounced granular incrustation.

Description: The basidiomata have a pileate-stipitate morphology and are small. The pileus is 23 mm in diameter, convex, and orange-brownish (5B5) to cinnamon-brown (6D6) in color; it has a furfuraceous surface that shows very fine scales at maturity, is slightly viscid how wet, and has entire margin. The hymenophore is adhered, with pores that are 0.8–1.2 mm in diameter, rounded when young, and irregular, angular or hexagonal at maturity; its color ranges from yellow (4A8) to gold; the tubes are 3–6 mm in length and concolorous to the pores; the color does not change when cut or changes from cinnamon (6D5) to ochraceous (6F4); and the hymenium is easily removable from the context. The context is 5 mm thick and a pale yellow color that changes to brownish orange (5C4) when cut; the context of the stipe is concolorous the pileus context. The stipe is 27 × 5 mm and cylindrical, its color is brown (5B4) to yellow-brownish (5B5), and the surface is furfuraceous to distinctly longitudinally streaked. The basal mycelial is citrus yellow (4A8). The odor is indistinctive, and the taste is also indistinctive. Chemical reaction: after applying KOH, the surface of the pileus and stipe becomes brown (5F5) to amber (6F8), while the hymenophore becomes brown-orange (5E7).

The basidiospores are 9–11(−14) × 4 −5 (−6) µm, (n = 30, Q = 2.2), smooth, and cylindrical to fusoid; they become yellow-greenish in KOH, with visible or no visible suprahilar depression, and amyloid in Melzer’s solution. The basidia are 22–30 (–38) × 8–10 (–12) µm, hyaline in KOH, clavate to subclavate in shape, and tetrasporic; they become olivaceous with content in Melzer’s solution. The hymenophoral trama is bilateral, of the *Boletus* type, and moderately divergent; the lateral stratum of the hyphae measuring 11–18 µm in diameter is cylindrical to subclavate, with content in Melzer’s solution, and has a thick wall; the middle stratum of the hyphae is 1–4 µm and cylindrical, with a gelatinous wall, and becomes hyaline in KOH. The pleurocystidia are 25 −40 (−68) × 6 −9 (−13) µm, fusoid to clavate in shape, hyaline in KOH, olivaceous with granulose content in Melzer’s solution, and thick-walled (1 µm). The cheilocystidia are 43 −60 (73) × 7 −10 (−14) µm and clavate to fusoid in shape, with very marked content at the apex, and have a thick wall (1–1.5 µm); they become hyaline in KOH and olivaceous in Melzer’s solution. The pileipellis is composed of epicutis with concatenated hyphae and cylindrical terminal cells; it is clavate to isodiametric in shape, measuring 20 −37 (−47) × 7 −12 (−17) µm, and becomes hyaline in KOH and olivaceous in Melzer’s solution with contents. The caulobasidia are 25 −40 (−55) × 7 −11 (−14) µm, clavate to mucronate, hyaline in KOH, olivaceous with contents in Melzer’s solution, and thick-walled (1–1.5 µm). The caulobasidia are 30 −35 × 8 −10 µm, clavate to subclavate in shape, tetrasporic, and concolorous to the caulobasidia. There are no clamp connections.

**Habit, habitat, and distribution:** This species shows a solitary growth pattern in *Quercus* forests and is associated with *Quercus scytophylla*; currently, it is only known from the type locality in Mixistlán, Oaxaca, Mexico.

**Notes:** *Aureoboletus ayuukii* differs from the rest of the species by its small basidiomata, ornamented pileous surface, and furfuraceous surface with very fine scales at maturity; its stipe has distinctly longitudinally streaks; the basidiospores are 9 −11(−14) × 4 −5 (6) µm and amyloid, which is not a common characteristic in Boletaceae; the pileipellis is formed by an epicutis. The phylogenetic analysis showed that *Aureoboletus ayuukii* is sister to the *A. formosus*, *A. longicolis*, and *A. singeri* species, with 1BI/75ML support. Taxonomically, *A. ayuukii* is similar to *A. miniatoaurantiacus*, which was found in China and described by Wu et al. [1], but also differs by its medium basidiomata, tomentose pileus surface, characteristic of being slightly viscid when wet, and sunflower yellow to maize yellow color; furthermore, the surface of its stipe has distinctly longitudinally streaks or dotted scales, and it has broad reticulations and short basidiospores compared to the other species described above at (6.5 −) 7 −11 (−11.5) × 4.5 −6 µm. Taxonomically, *A. ayuukii* shares some characters with *A. auriflammeus* found in USA and Mexico, but it differs by having a pileus surface that is tomentose to pulverulent, an orange to brown-orange color, a fibrillose stipe surface that is whitish to pale yellow, and basidiospores that are 9–10 (−13) × 3 −4 (−) µm [1,13], and *A. auriflammeus* is distributed in cloud forests, while *A. ayuukii* is distributed in *Quercus scytophylla* forests.

**Figure 3:**
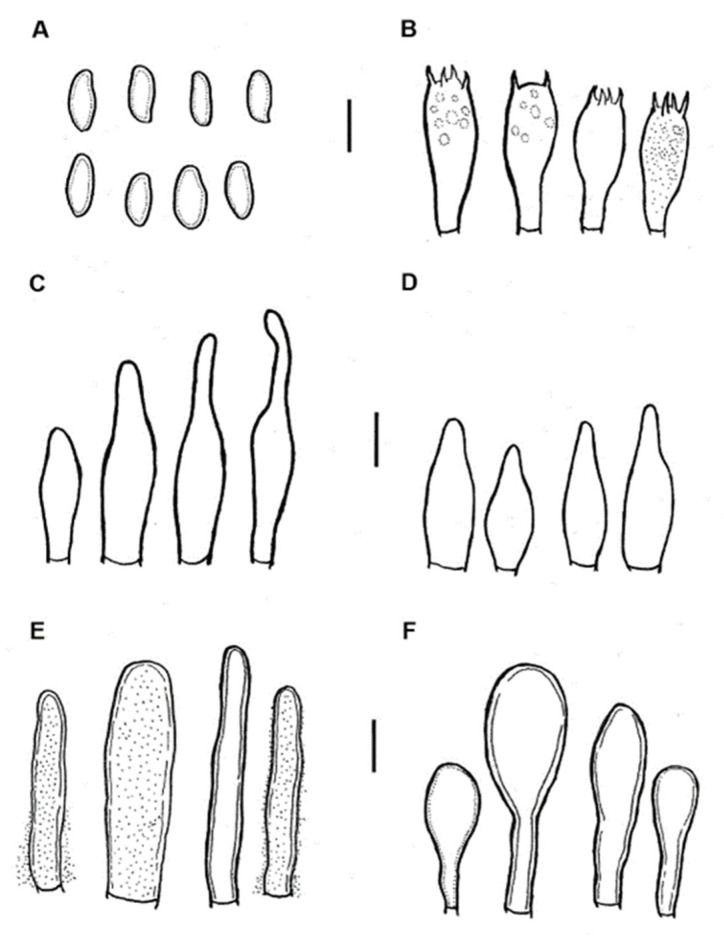
*Aureoboletus elvirae* (794-ITCV, MEXU-HO-HO 29006, holotype): (**A**) basidiospores; (**B**) basidia; (**C**) cheilocystidia; (**D**) pleurocystidia; (**E**) pileipellis; and (**F**) caulobasidia. Scle bars: 10 µm.

***Aureoboletus elvirae*** Ayala-Vásquez, García-Jiménez & de la Fuente, sp. nov. (Figure 3).

MycoBank no: MB 834539.

Etymology: It is named in honor of Elvira Aguirre-Acosta, an eminent Mexican mycologist and an extraordinary human being, who is a curator of the collection of mushrooms of MEXU-HO at the National Autonomous University of Mexico for more than 49 years, and an expert in Agaricales and Gasteroid fungi.

Holotype: Mexico, Oaxaca state, Mixistlan de la Reforma Municipality, Santa Maria Mixistlan Town, Paaxoom place, 2211 m.a.s.l., 17°08′18.28″ N 96°05′17.64″ W, in *Quercus scytophylla* forest, 4 November 2016, (794-ITCV, MEXU-HO-HO 29006, LSU GenBank Num: OQ975746, *RPB2* GenBank Num: OQ938893).

Diagnosis: This species has small basidiomata that are honey to brown-orange in color. The pileus is broadly convex and mamelonate to umbonate, and the surface has sharp, small triangular scales and crenulate margin tht is somewhat decurved; the hymenophore has angular to circular pores; the basidiospores are (7 −) 8 −10 (−12) × 4 −5 (−6) µm and elliptical; and the pileipellis is formed by trichoderm, with thick-walled terminal cells.

Description: The pileus is 27 mm in diameter, convex, mamelonate to umbonate, and honey, golden-brown (5D7) to yellowish red (8A6) in color, with sharp, small triangular scales and a crenulate margin that is decurved. The hymenophore is adnexed, with pores that are 1–1.5 mm in diameter; circular to angular in shape; and whitish orange (6A2) to pale orange (6A3) honey in color; its tubes are 12 mm long and concolorous to the pores. The context is 6 mm thick and whitish to pale yellow (4A4) in color, while the stipe context is pale-yellow to orange-brown (5A3). The stipe is 29 × 6 mm, subclaviform, and brown-orange (5A5) to honey in color, with its surface being furfuraceous to striated. The mycelium is white. The odor is fungoid. The taste is like that of raw potato. Chemical reaction: The pileus and context turn dark brown (5F8) in KOH, whereas the stipe turns reddish brown (7E8). The basidiospores are (7 −) 8 −10 (−12) × 4 −5 (−6) µm, (n = 35, Q = 1.9 −2.1), and elliptical; the inside view is inequilateral with an obtuse apex, and the central view is oblong to ovoid with or without suprahilar depression; they turn yellow-green in KOH and olive green in Melzer’s solution. The basidia are 21 −25 (−30) × 6 −10 (−11) µm, claviform, and 4-spored; they turn hyaline in KOH and greenish with content in Melzer’s solution. The pleurocystidia are 33 −41 × 7 −10 µm, very scarce, fusoid, and thick-walled. The cheilocystidia are 24 −43 × 9 −11 µm and fusoid-ventricose, with some being clavate at the apex, somewhat mamillated to rounded, thick-walled. The hymenophoral trama is divergent and of the *Boletus* type; the middle stratum of the tubulous hyphae is 3 −8 µm in diameter, and some have gelatinous walls; the lateral stratum of the hyphae is 5–14 µm in diameter, and turns hyaline to yellowish in Melzer’s solution. The pileipellis is formed by trichoderm and is up to 150–180 µm thick, with terminal cells that are 14 −45 (−55) × 6 −9 (−12) µm; it is cylindrical to subclavate in shape, turns hyaline to yellowish in KOH, is covered with bright yellow extracellular crystals that are visible in Melzer’s solution, and is thick-walled. The stipitipellis is 70–90 µm thick, with caulocystidia that are 14 −45 (−55) × 6 −9 (−12) µm and claviform, with some being napiform; it is thick-walled and turns hyaline in KOH and olive in Melzer’s solution with visible content. The clamp connections are absent.

**Habitat and distribution**: This species shows a solitary growth pattern in *Quercus* forest during November and is associated with *Quercus scytophylla*; it is known from Sierra Norte, Oaxaca, Mexico.

**Notes**: *Aureoboletus elvirae* is distinguished from other species by having basidiomata that are honey, golden-brown to yellowish red in color, pileus that is convex to mamillated, and surface with very sharp triangular scales. Microscopically, it is characterized by having elliptical basidiospores, fusoid-ventricose or clavate cystidia, and a pileipellis that is formed by trichoderm with thick-walled cells. Morphologically and phylogenetically, *A. elvirae* is included in clade II nested near *A. catenarius* G. Wu & Zhu L. Yang, *A. citriniporus* (Halling) Klofac, *A. moravicus* (Vaček) Klofac, *A. roxanae* (Frost) Klofac, and *A. yunnanensis* G. Wu & Zhu L. Yang, according to Zhang et al. [8]. *Aureoboletus roxanae* has only been found in North America, *A. moravicus* has been found in Europe, and the other three species are from China. *Aureoboletus elvirae* differs from *A. roxanae* by the ornamentation of the surface of its pileus, its honey-colored context and hymenophore, and is narrow base of the stipe, while *A. roxanae* has the stipe that is clavated with a bulbous base and the context is whitish. *Aureoboletus elvirae* is a rare species since only one specimen was found, which is associated with *Q. scytophylla*, during 10 years of regular explorations in *Quercus* forests. Meanwhile, *A. roxanae* is more commonly distributed in cloudy forests, with a phenology from July to October.

**Figure 4:**
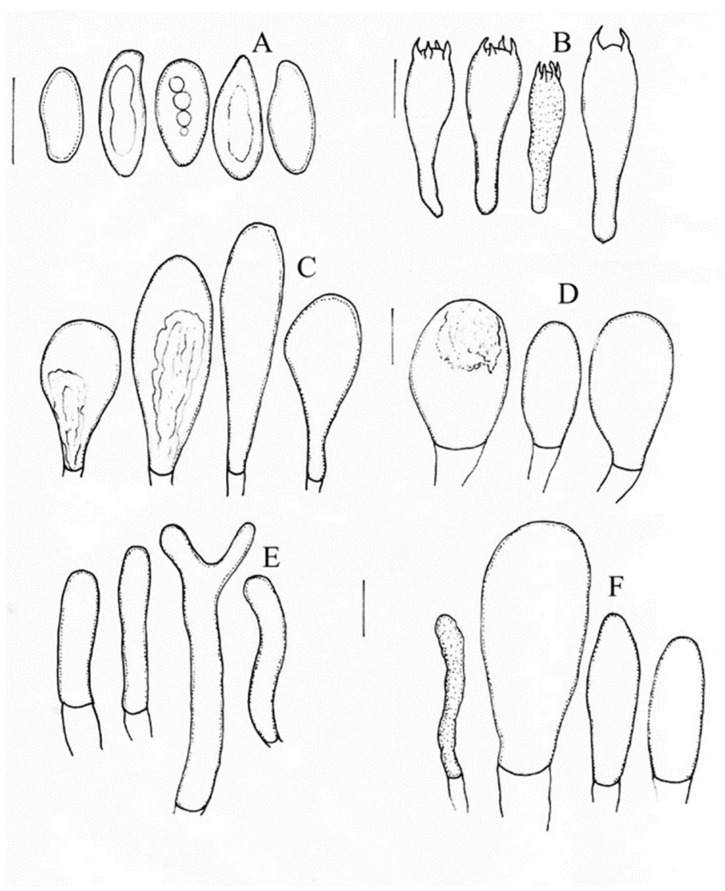
*Aureoboletus readii* (MEXU-HO 30443, holotype): (**A**) basidiospores, (**B**) basidia, (**C**) pleurocystidia, (**D**) cheilocystidia, (**E**) pileipellis, and (**F**) caulocystidia. Scale bars: 10 µm.

**Figure 5:**
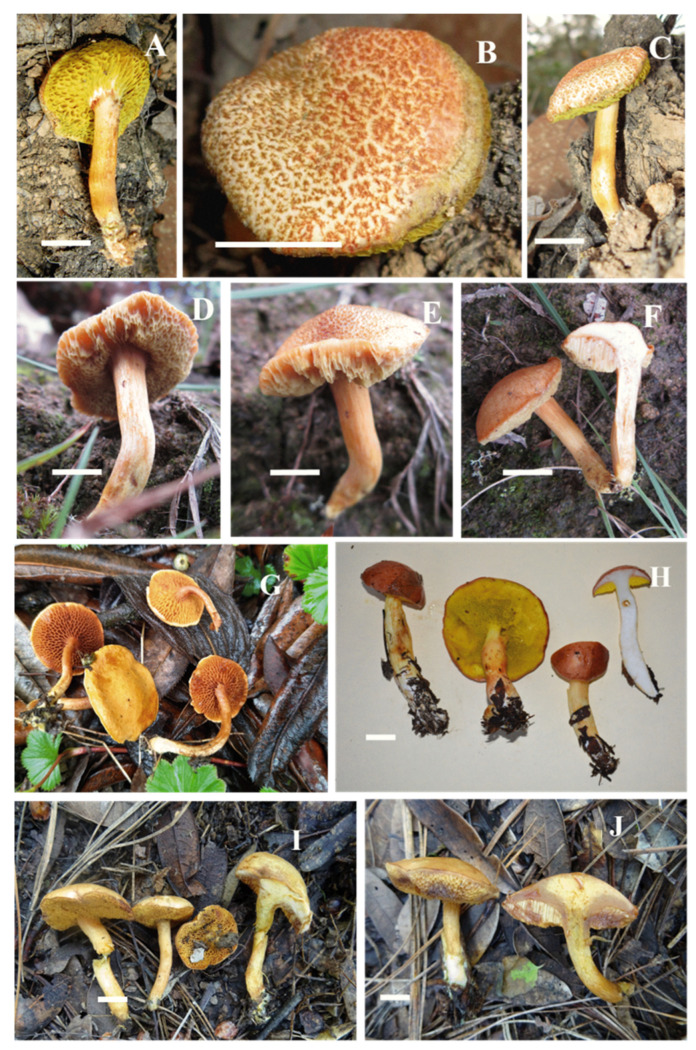
Fresh basidiomata. (**A**–**C**) *Aureoboletus ayuukii* (MEXU-HO HO-30448); (**D**–**F**) *Aureoboletus elvirae* (MEXU-HO 29006); (**H**) *Aureoboletus readii* (MEXU-HO 30443, holotype); (**G**) *Chalciporus perezsilvae* (MEXU-HO 30459, holotype); (**I**–**J**) *Chalciporus piedracanteadensis* (MEXU-HO 30436, holotype). Scale bars (**A**–**I**) = 10 mm. Credit pictures: (**A**–**G**,**I**–**J**) Olivia Ayala-Vásquez, and (**H**) Jesús Pérez-Moreno.

***Aureoboletus readii*** Ayala-Vásquez, Pérez-Moreno, Martínez-Reyes, Carbajal-Ramírez, sp. nov. (Figure 4 and Figure 5).

MycoBank number: MB 850014

Etymology: This species is named in honor of Sir David Read, who is an eminent mycorhizologist, an Emeritus Professor at the University of Sheffield, and the life mentor of the corresponding author, with more than 55 years of experience studying mycorrhizal fungi; Sir David Read is also the Secretary of the Royal Society (London), who was knighted in 2007 by the Queen of England for his scientific contributions, and an extraordinary human being.

Holotype: MEXICO, Estado de Mexico, Ocuilan Municipality, San Juan Atzingo Town, 17°08′38″ N, 96°06′22″ W, alt. 2043 m, 15 July 2021, Carbajal-Ramírez E., Pérez-Moreno J, Ayala-Vásquez O., CP48 (MEXU-HO 30443, holotype).

Diagnosis: This species has medium-to-small basidiomata. The pileus surface is very viscid, the hymenophore is adhered, and the pores are circular. It is gold in color, the stipe is subclavate, the furfuraceous surface has superficial longitudinal streaks, the basidiospores are 10−)11−13 (−14) × (3.5−) 4−5 µm.

Description: Pileus is 46 –84 mm diameter, broadly convex or plane-convex, and red, reddish-brown, or red-cinnamon; its surface is very viscid, and the margin is slightly serrate, curved when young, and incurved and slightly serrate at maturity. The hymenophore is adhered, with pores that are 0.5−0.1 µm, circular to irregular in shape, and golden in color; pores are 0.5 mm in the center of the hymenophore and >5 mm in its margin, the tubes are 6−7 mm and concolorous to the pores. The context is 5−7 mm and whitish, and does not change color when cut. The stipe is 55 −120 × 15−18 mm and subclavate and has a furfuraceous surface; it is white when young and turns pale red when mature, especially in the middle part and base; there is a high abundance of mycelia in the middle part of the stipe, which are whitish in color. The odor is fungoid. The taste is also fungoid. The basidiospores are (10−)11−13 (−14) × (3.5−) 4−5 µm, (n = 40, Q = (2.5−) 2.7 (−2.8) µm), smooth, and cylindrical; they turn hyaline in KOH and pale brown in Melzer’s solution. The basidia are (27−) 28−34× (10−) 11−12 µm, clavate, thin-walled, and tetrasporic, with 2–4 µm sterigmata; they turn hyaline in KOH and pale yellow in Melzer’s solution. The pleurocystidia are (35−) 38−40 (−50) × (10− 13−16 (−21) µm, claviform to piriform in shape, with basal septa (1–2), and thick-walled (1–2 µm); they turn hyaline to pale yellow in Melzer’s solution. cheilocystidia 27−50 × 12−15 µm, clavate, with a rounded apex; they turn hyaline in KOH and have visible granular content in Melzer’s solution. The hymenophoral trama is divergent and of the *Boletus* type; the middle stratum of the tubulous hyphae is 4–11 µm in diameter with gelatinous walls; the lateral stratum of the hyphae is 3–9 µm in diameter and turns hyaline in KOH and yellowish in Melzer’s solution. The pileipellis is formed by ixotrichoderm; the prostrate hyphae turn hyaline in KOH, and the terminal hyphae are (30−) 45−60 (−110) × (5−) 6−7 (8) µm, cylindrical, and thick-walled.

Additional specimens examined: MEXICO, Estado of Mexico, Ocuilan Municipality, San Juan Atzingo Town, 17 September 2021, Martínez-Reyes M., Carbajal-Ramírez E., CP123-1(MEXU-HO 30439); 18 September 2021, Martínez-Reyes M., Carbajal-Ramírez E., CP123-2, (MEXU-HO 30440), 19 September 2021, Martínez-Reyes M., Carbajal-Ramírez E., CP124-1, (MEXU-HO 30441); and 20 September 2021, Martínez-Reyes M., Carbajal-Ramírez E., Ayala-Vásquez O., CP124-2, (MEXU-HO 30442).

**Habit, habitat, and distribution:** This species shows a solitary and disperse growth pattern in mixed forests, and is putatively ectomycorrhizal with *Quercus laurina*; currently, it is only known from San Juan Atzingo, Estado of Mexico, Mexico.

**Notes:** *Aureoboletus readii* belongs to the *auriporus* complex. *A. auriporus* differs from *A. readii* by having a pileus surface that is very viscid, a color that is reddish-brown to red-cinnamon, and basidiospores that are (10−)11−13 (−14) × 3.5 µm. The pileus of *A. auriporus* is glabrous, pruinose to tomentose, yellowish-brown to grayish-brown, and 11−16 × 4−6 µm, Q = 2.8 µm, and it is distributed in USA, from New Jersey and Florida west to Mississippi and Texas [35]. While *A. pseudoauriporus* has a pileus surface that is glabrous and pinkish to pinkish red and basidiospores that are (14–)15–17(–18) × 5–6.5 µm, Q = 2.79 [11], *A. gentilis* has a pileus that is shiny, viscid, and pinkish-brown and basidiospores that are (11−)12−5(−17) × (4.5−)5−6.5 µm, Q = 2.4 µm, and it only has been recorded from Europe [36].

The micromorphology of the newly described species belonging to the genus *Aureoboletus* including *A. ayuuki*, *A. aureoboletus*, *A. readii* and to the genus *Chalciporus* including *C. perezsilvae* and *C. piedracanteadensis* are shown in Figure 5.

*Chalciporus* Bataille

Description: This species has small basidiomata with a stipitate-pileate morphology and a tubular hymenophore. The pileus is convex to broadly convex, glabrous to obscurely subtomentose, and dry, but sometimes subviscid when wet; its color ranges from yellow, brown-orange, and ochraceous to red-brown; the context is whitish to light yellow, with the color unchanging or staining bluish slowly when injured. The hymenophore is decurrent, and the pores and tubes are concolorous; the color ranges from pinkish red, yellow, and ochraceous to reddish brown, and it remains unchanging in color or stains bluish to dull blue slowly when cut. The pileipellis is composed of trichoderm with matted interwoven hyphae. The pleurocystidia and cheilocystidia are subfusiform-ventricose. The basidiospores are smooth, subfusiform, and pale yellow to olive-yellow in color. The clamp connections are absent [25]. The type species is *Chalciporus piperatus* (Bull.) Bataille.

Key to species of *Chalciporus* in Mexico
1a. Red, brown-reddish, brown-orange, cinnamon to ferruginous basidiomata …….……………………………………………………………………………………………………….. 21b. Yellow, ochraceous-yellow to yellow-brown basidiomata; yellow, gold to yellow-brownish hymenophore; and (7−) 8−10 (−11) × (2.5−)3−3.5 (−4) µm basidiospores ………………………………………………………………………………………………………………………………………………………………………… *Chalciporus piedracanteadensis*2a. Pores < 1.5 in diameter, and circular to angular ……………………………………………………………………………………………………………………………………………… 32b. Pores is 1.5 mm in diameter, and cinnamon, ochraceous, or ferrugineus; pileus is 20− 60mm, with slightly viscid to dry surface, context is whitish-yellow to pink; 7.2−10.4 × 2.8−4 µm basidiospores; and the taste is very spacy…………………………………………………………………………………………………………………….. *Chalciporus piperatus*3a. Small to medium basidiomata that are 20–45 mm; surface of pileus is dry, slightly velvety when young and becoming finely cracked with age, while the color is red or reddish when young, and becomes yellower with age; pores are 0.5 mm in diameter; basidiospores are 10−14.5 (−16.5) × 3−4.5(−4.8) µm ………………………………………………………………………………………………………………………………………………………………………………….. *Chalciporus rubinellus*3b. Small basidiomata that is 20–30 mm; surface of pileus is glabrous, dry, entirely marginal when young, and somewhat irregular at maturity, the diameter is 0.3–0.8 mm, the shape is circular to hexagonal, and the color is pale orange, red-brown to cinnamon, unchanging when pores are bruised; basidiospores are 9–11× 3–4 (–5) µm; and this species is distributed in cloud forest……………………………………………………………………………………………………………………………………… *Chalciporus perezsilvae*

**Figure 6:**
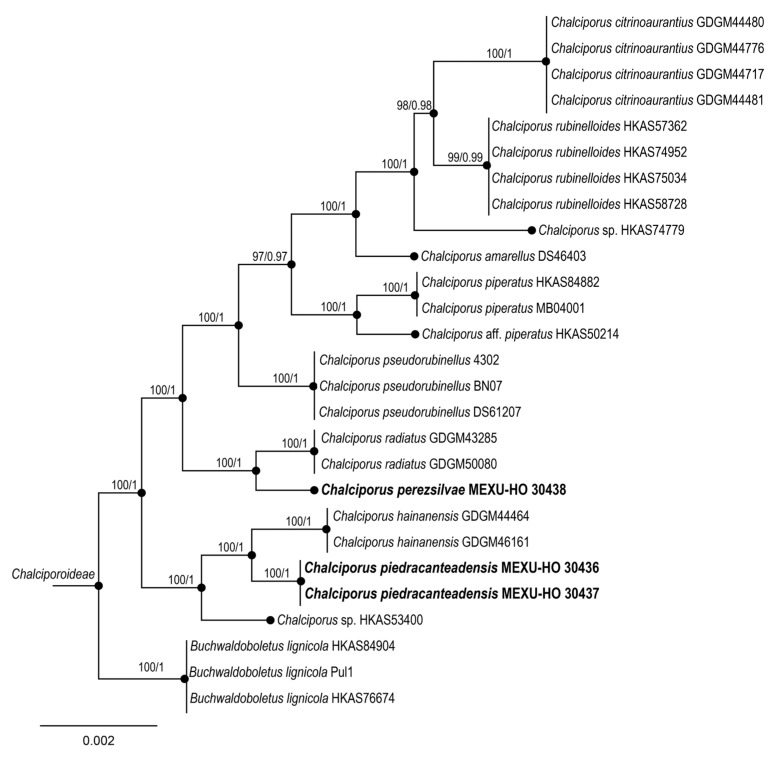
Maximum likelihood phylogenetic analysis based on the concatenated ITS, nLSU, *rpb2*, and tef sequence alignments. Maximum parsimony and Bayesian analyses recovered identical topologies with respect to the relationships among the main clades of *Chalciporus*. For each node, the following values are provided: maximum parsimony bootstrap (70%)/and posterior confidence (0.90 *p*-value). The scale bar represents the expected number of nucleotide substitutions per site.

**Figure 7:**
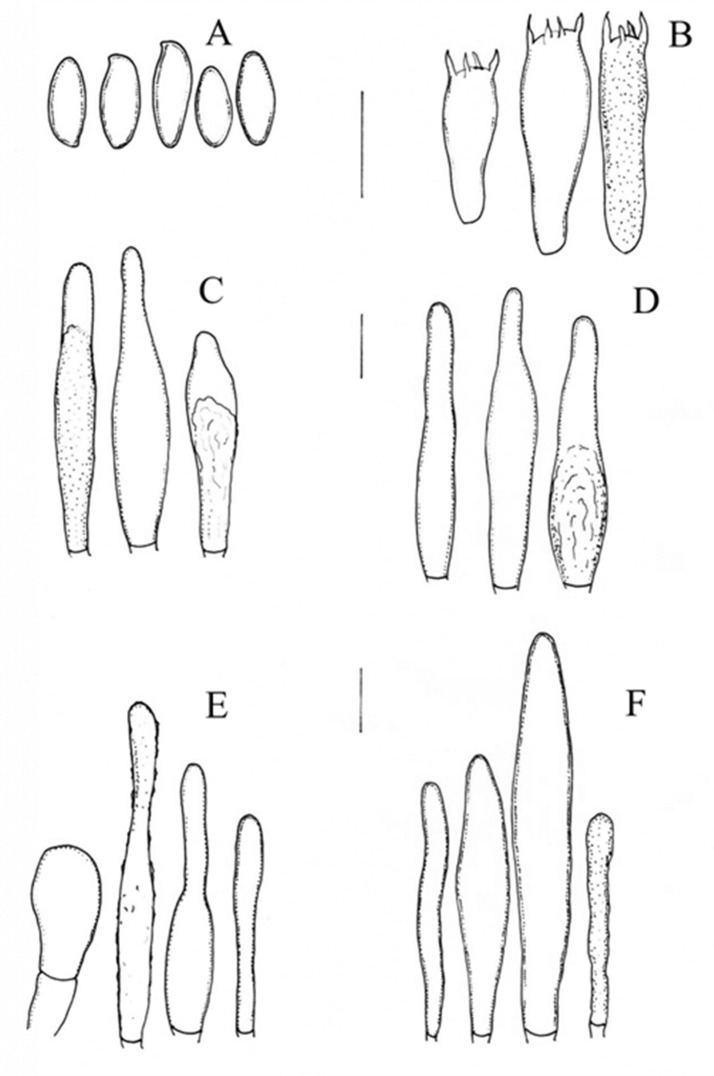
*Chalciporus perezsilvae* (MEXU-HO holotype): (**A**) basidiospores, (**B**) basidia, (**C**) pleurocystidia, (**D**) cheilocystidia, (**E**) pileipellis, and (**F**) caulocystidia. Scale bars: 10µm.

***Chalciporus perezsilvae*** Pérez-Moreno J., Ayala-Vásquez O., Martínez-Reyes M., Martínez-González C. Figure 5, Figure 6 and Figure 7.

MycoBank number: MB840856

Etymology: The name is in honor of Dr. Evangelina Pérez Silva, a pioneer woman of Mexican mycology and a specialist in Ascomycetes and the genus *Inocybe*, who has inspired and supported many generations of mycologists during her four decades of scientific career.

Holotype: MEXICO, Oaxaca, Santa María Tlahuitoltepec Municipality, Santa María Yacochi Town, Zempoaltepetl Hill, cloud forest, 27 June 2022, Ayala-Vásquez O, Martínez-Reyes M., Martínez-Gonzalez CR., 1571, (MEXU-HO 30459).

Diagnosis: This species has small basidiomata with a pileate-stipitate morphology. The hymenium has circular to hexagonal pores, and the color is pale orange and red-brown to cinnamon.

Description: The pileus is 20–30 mm, convex to plane in shape, and light orange (5A6), golden yellow (5B8), or brownish-yellow (5CA) in color; it has a glabrous, dry surface that is entirely marginal when young, and somewhat irregular at maturity. The hymenophore is subdecurrent and tubular, with pores that are 0.3–0.8 mm; it is circular to hexagonal in shape, and its color ranges from pale orange (5C7) and red-brown (6D8) to cinnamon (7E8) and brown (5D8), which remains unchanged when bruised; the tubes are 4–6 mm length and concolorous to the pores, which remain unchanged when cut. The context is 4–6 mm length; the pileus context is pale light orange (5A6) that remains unchanged when cut, while the context of the stipe is compact and yellow-brown (5D8) or greyish-orange (6B5) in color. The stipe is 30−40 × 3−5 mm, cylindrical, reddish-brown (7A6) at the apex, cinnamon (7E8) at the middle part, and yellow (5B7) to yellow-brown (5D6) at the base; the stipe has a finely fibrillose to furfuraceous surface. The taste is spicy. There is no odor.

The basidiospores are 9−11× 3−4 (−5) µm, (Q = (n = 33, 1.5) 1.7 (1.8) µm), elongate to cylindrical in shape, and smooth, with suprahilar depression and a thin wall; they turn hyaline in KOH and yellowish in Melzer’s solution. The basidia are 20−25 × 7−9 µm and clavate, with four sterigmata; they turn hyaline in KOH and yellow in Melzer’s solution. The pleurocystidia are 20−45 × 5−7 (−11) µm, fusoid to mucronate, and thick-walled; they turn hyaline in KOH and the content is pale brown in Melzer’s solution. The cheilocystidia are (30−) 42−60 × (4) 5−8 µm, subfusoid, ventricose-fusoid or clavate in shape, and thick-walled; they turn hyaline to pale yellow in KOH and yellow in Melzer’s solution. The hymenophoral trama is subparallel and composed of a central stratum with tubulose hyphae that are 3–10 µm in diameter, with gelatinized walls; it is hyaline and thin-walled; the lateral stratum is composed of tubulose hyphae, 7–12 µm in diameter, hyaline, and thin-walled. The stipitipellis formed by an arrangement of vertical tubular, cylindrical hyphae with a few caulocystidia that are (22−) 42−66× (6−) 10−15 µm in diameter; it turns hyaline to light yellow in KOH. The pileipellis is formed by ixotrichoderm with interwoven hyphae that are 2−5 µm, is cylindrical, and has a gelatinous wall; it turns pale yellow in KOH and golden in Melzer’s solution.

**Habit, habitat, and distribution**: This species has a disperse growth pattern in cloud forests; it is currently recorded only from Zempoaltepetl Mountain, Oaxaca, Mexico.

**Notes**: *Chalciporus perezsilvae* is characterized by small basidiomata, pores that are circularly to radially arranged, and a short stipe compared to other species; the basidiospores are 9−11× 3−4 (−5) µm. *Chalciporus chontae* differs from *C. perezsilvae* by having pores that are small to medium in size and circularly arranged, and a stipe that is cylindrical to subclavate in shape; *C. radiatus* has a pileus with a velvety and tomentose surface, with a color ranging from greyish orange and greyish-red to greyish brown and brownish orange; its hymenophore is light orange, yellowish, brown, or reddish brown in color; the basidiospores are 7–8 × (3–) 3.5×4 mm and fusoid, cylindrical, or oblong in shape [37].

**Figure 8:**
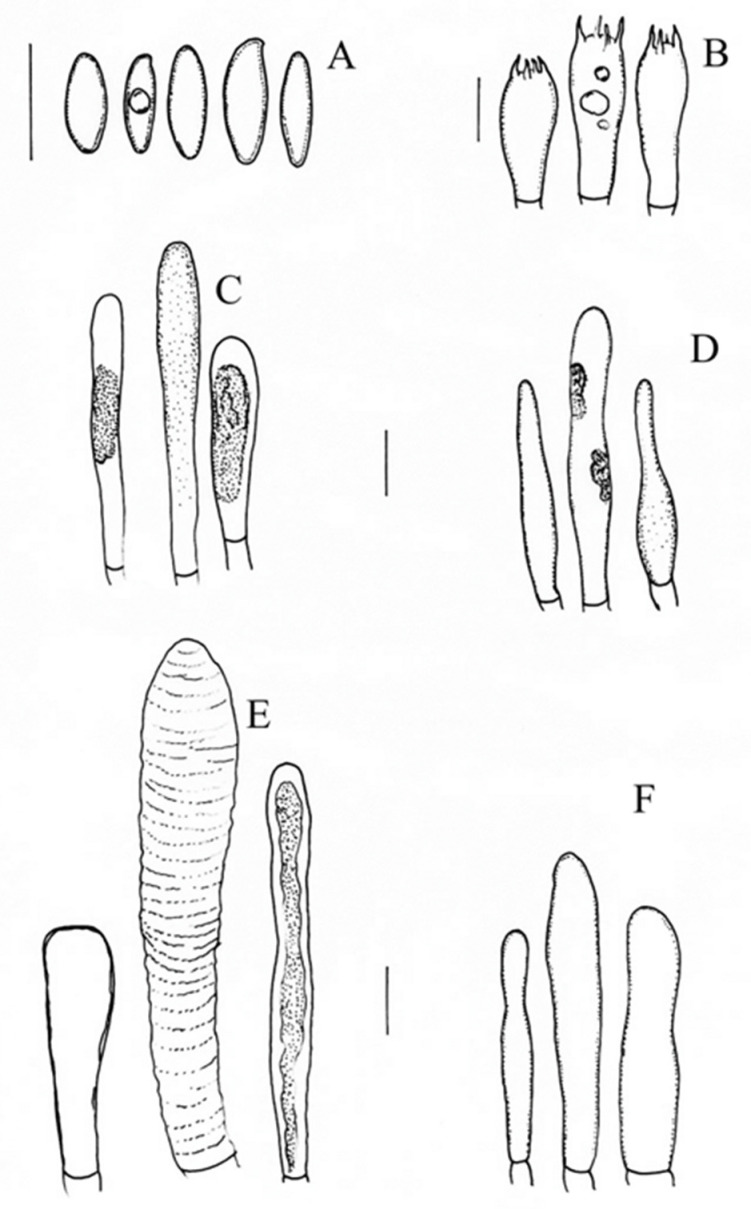
*Chalciporus piedracanteadensis* (MEXU-HO 30436, holotype): (**A**) basidiospores, (**B**) basidia, (**C**) pleurocystidia, (**D**) cheilocystidia, (**E**) pileipellis, and (**F**) stipitipellis. Scale bars: 10 µm.

***Chalciporus piedracanteadensis*** Ayala-Vásquez, Pérez-Moreno, Martínez-Reyes, sp. nov. Figure 5 and Figure 8.

MycoBank number: MB 850,032

Etymology: The name *piedracanteadensis* refers to the distribution site of the holotype, *Piedra Canteada*, where different mycological projects have been conducted with the permanent support from local communities and the Mexican Government.

Holotype: MEXICO, Tlaxcala, Nanacamilpa Municipality, San Felipe Hidalgo Town, Reserve Piedra Canteada, El Plano place, 23 August 2021, Ayala-Vasquez O., 1560, (MEXU-HO 30436).

Diagnosis: The basidiomata are small and ochraceous-yellow to yellow-brown in color; the hymenophore’s color ranges from yellow to gold when young, and yellow-brownish to rust when mature, and it changes to an amber or honey color when cut; the basidiospores are (7−) 8−10 (−11) × (2.5−)3−3.5 (−4) µm.

Description: The basidiomata are small and have a pileate-stipitate morphology. The pileus is 22−23 mm in diameter, convex, and ochraceous to yellow-brownish (5B5) in color; it has a furfuraceous surface and is slightly viscid how wet, with entire margin. The hymenophore is adhered and rounded when young, but irregular to hexagonal when mature; the pores are 0.8−1.2 mm in diameter, yellow to gold when young, and yellow-brownish (5B5) to rust when mature; the tubes are 3 mm long and concolorous to the pores, and their color changes to cinnamon to ochraceous when cut; the hymenium is easily removable from the context. The context is 5 mm thick and pale yellow, and the color changes to brownish-orange (5C4) to cinnamon when cut; the context of the stipe is concolorous to the pileus context. The stipe is 27−× 5− mm, cylindrical, and brown (5B4) or yellow-brownish (5B5), and it has a furfuraceous surface. The basal mycelium is citrus yellow. The odor indistinctive, and the taste is also indistinctive.

The basidiospores are (7−) 8−10 (−11) × (2.5–)3– 3.5 (–4) µm, (n = 45, Q = 2.6–2.8–3 µm), ellipsoidal to cylindrical in shape, with suprahilar depression, and thick-walled; they turn yellow in KOH and yellowish-brown in Melzer’s solution. The basidia are (18–) 20–25 (–32) × (6–)7–9 µm and clavate and have four sterigmata; they turn hyaline in KOH and yellow in Melzer’s solution. The pleurocystidia are 33–40 × (8–)9–12 µm, subclavate, subfusoid to mucronate, and thin-walled; they turn hyaline to pale yellow in KOH and yellow in Melzer’s solution. The cheilocystidia are 40–45 × 7.5–10 µm, lageniform, fusoid, and thin-walled; they turn hyaline to pale yellow in KOH and yellow in Melzer’s solution. The hymenophoral trama is subparallel, composed of a central stratum with tubulose hyphae, which are 2–8 µm in diameter, with gelatinized walls, hyaline, and thin-walled; the lateral stratum is composed of tubulose hyphae, 9−11 µm in diameter, hyaline, and thin-walled. The stipitipellis contains a layer of repent to suberect branching hyphae, 5–10 µm in diameter; the terminal elements are cylindrical to subclavate and turn hyaline in KOH, and golden in Melzer’s solution. The pileipellis is formed by trichoderm with prostrate hyphae of 28−50 × 10−12 µm, cylindrical, subclavate to truncate, and thin-walled; it turns pale yellow in KOH and golden in Melzer’s solution.

**Habit, habitat, and distribution:** This species shows a solitary growth pattern in *Quercus-Pinus* forests and mixed coniferous forests, and is putatively associated with *Pinus pseudostrobus*; currently, it is only known from the region of Piedra Canteada, Tlaxcala, Mexico.

**Additional specimens examined:** MEXICO, Tlaxcala, Nanacamilpa Municipality, San Felipe Hidalgo Town, Reserve Piedra Canteada Place, 4 September 2021, Ayala-Vásquez O. 1561, (MEXU-HO 30437); 4 October 2021, Ayala-Vásquez O., Martínez-Reyes M., 1561, (MEXU-HO 30438), 17 September 2022, Ayala-Vásquez O., (CP1570).

**Notes:** According to diagnosis, the genus *Chalciporus* has a hymenophore that is pinkish red to reddish brown. Nevertheless, *Chalciporus piedracanteadensis* differs from other species by having basidiomata that are ochraceous-yellow to yellow-brown; a hymenophore that is yellow to gold when young, and yellow-brownish to rust when mature; a context that is pale yellow, which changes to brownish-orange to cinnamon when cut. According to our phylogenetic analysis, the closest species to *Chalciporus piedracanteadensis* is *C. hainanensis* from China [16], but differs by having basidiomata that are grayish yellow, olive yellow, grayish orange to brownish orange in color; a context that is white to yellowish white, which changes to blue at first and then gradually changes to grayish orange, brownish orange, or grayish red when bruised; this species grows in forests of Fagaceae [16], while *C. piedracanteadensis* is putatively associated with *Pinus teocote* and *P. pseudostrobus.*

## 4. Discussion

Among the new species described in this work, *Aureoboletus readii* is consumed by members of the Tlahuica-Pjiejkakjoo culture, who are located in central Mexico, thus augmenting the species number consumed in the country to five. Previously, *Aureoboletus auriflammeus*, *A. auriporus*, *A. betula*, *A. gentilis*, *A. longicolis*, *A. mirabilis*, *A. moravicus*, *A. projectellus*, *A. russellii*, *A. shichianus*, and *A. thibetanus* have been recorded to be edible [4]; therefore, currently, 11 species in the genus are known worldwide to be used as a food source.

*Aureoboletus auriporus* (Peck) Pouzar represents a species complex [11]. In our phylogenetic analysis, *A. readii* belongs to the *A. auriporus* clade, together with *A. pseudoauriporus* and *A. gentilis*, which are the type species of the genus. *Aureoboletus readii* differs from *A. psedoauriporus* due to its non-viscid pileus that is pink to pinkish red, and its basidiospores that are (14–)15–17(–18) × 5–6.5 µm, whereas *A. readii* has a pileus surface that is very viscid and red, reddish-brown, or red-cinnamon in color, as well as short basidiospores of (10–)11–13 (–14) × 3.5 µm, compared to *Aureoboletus auriporus* basidiospores that are 11–16 × 4–6 µm [35] from the type collection. Meanwhile, *A. ayuukii* has sister species: *A. formosus* from China [38], *A. longicolis* also from China [38], and *A. singeri* from Mexico. Phylogenetically, *A. elvirae* is a sister group to *A. roxanae*, with support of 0.99BI/84ML, although our phylogram shows that *A. roxanae* is probably polyphyletic. *Chalciporus* is a genus that has received little attention in Mexico; thus far, only *C. piperatus and C. rubinellus* have been described with detailed morphological data in the north and the Gulf of Mexico [39]. This work describes two species from Central and Southeast Mexico. *Chalciporus perezsilvae* is from cloudy forests in Southeast Mexico. According to our phylogram, *C. perezsilvae* is a sister group of *C. radiatus* that has been described by Xu et al. [37] in southern China, growing in *Cunninghamia lanceolata*, *Castanopsis* sp., and *Cyclobalanopsis* spp mixed forests. Meanwhile, *C. piedracanteadensis* is only known from Central Mexico in a mixed forest (with *Abies religiosa*, *Pinus pseudostrobus*, and *Quercus laurina*) and a *Pinus-Quercus* forest (with *Quercus laurina*, *Quercus* aff. *crassifolia*, *Pinus teocote*, and *Pinus montezumae*). This species is a sister group of *Chalciporus hainanensis* Ming Zhang & T.H. Li, which has been described by Zhang et al. [16] from Hainan Province, China, under Fagaceae.

## 5. Conclusions

Boletaceae includes a large biodiversity which has not been completely described yet. Particularly, a few genera have received little attention despite their paramount ecological importance in the structure and functioning of the forests where they grow. In the case of Mexico, *Aureoboletus* and *Chalciporus* are the genera that have been scarcely studied in an integrative way including macro-, micromorphological, molecular, and phylogenetic analyses. Additionally, some taxa have great biocultural importance for native cultures. In this work, we describe three and two new species of *Aureoboletus* and *Chalciporus*, respectively. Additionally, we record the edibility of *A. readii* among the Tlahuica-pjiekakjoo culture, where it is widely consumed and known by the local name of *Nchjo-panci*. It is, therefore, an urgent need to continue studying both the biodiversity of Boletaceae Mexican taxa and those species with biocultural importance since a large amount of knowledge is quickly disappearing due to a strong transculturation process.

## Figures and Tables

**Table 1:** GenBank accession numbers of the sequences used in the phylogenetic analyses of *Aureoboletus* in this study. Sequences in bold were generated in this study.

Fungal Taxa	Specimen Voucher	ITS	LSU	*RPB1*	*RPB2*	*TEF*
*A. auriflammeus*	CFMR BOS 699	----------------	MK601706	----------------	MK766269	MK721060
*A. auriporus*	MAN020	----------------	JQ003659	----------------	----------------	----------------
*A. auriporus*	BDCR0431	----------------	HQ161871	DQ534636	----------------	----------------
*A. auriporus*	strain 35/97	----------------	DQ534636	----------------	----------------	----------------
***A. ayuukii***	**Holotype MEXU 30448**	**OR421042**	**OR421569**	**OR50581**	**OR50752**	**OR50215**
*A. catenarius*	GDGM45142	----------------	MN204514	----------------	----------------	----------------
*A. catenarius*	HKAS54463	----------------	KY990509	KT990890	KT990348	KT990710
*A. catenarius*	HKAS54467	----------------	KT990510	----------------	KT990349	KT990711
*A. clavatus*	GDGM42962	----------------	KR052045	KR052056	----------------	----------------
*A. clavatus*	GDGM42963	----------------	KR052046	KR052057	----------------	KR052054
*A. clavatus*	GDGM42984	----------------	KR052047	----------------	----------------	KR052055
*A. clavatus*	HKAS59802	----------------	KR052044	----------------	----------------	KR052053
*A. duplicatoporus*	HKAS63009	----------------	KT990511	KT990891	KT990350	KT990712
*A. duplicatoporus*	HKAS83115	----------------	KT990512	KT990892	KT990351	KT990713
*A. duplicatoporus*	HKAS50498	----------------	KF112361	KF112561	KF112754	----------------
***A. elvirae***	**MEXU-HO 30703-ITCV794**	----------------	**OQ938893**	----------------	**OQ975746**	----------------
*A. formosus*	GDGM44441	----------------	NG057082	----------------	KF291751	----------------
*A. garciae*	Holotype MEXU 29006	----------------	MH337251	MT228982	MT228983	----------------
*A. garciae*	MEXU 30133	----------------	MT228976	MT228981	MT228984	----------------
*A. garciae*	MEXU 30134	----------------	MT228977	MT228980	MT228985	----------------
*A. garciae*	MEXU 30135	----------------	MT228978	MT228979	MT228986	----------------
*A. gentilis*	MG372a	----------------	KF112344	KF112557	KF112741	KF134014
*A. gentilis*	Pug1	----------------	DQ534635	----------------	----------------	KF030399
*A. griseorufescens*	GDGM28490	----------------	MH670278	----------------	MH700241	----------------
*A. griseorufescens*	ZM131	----------------	MH670279	MH700220	MH700242	----------------
*A. innixus*	CFMR BOS 544	----------------	MK601707	----------------	MK766270	MK721061
*A. innixus*	MB03 104	----------------	KF030239	----------------	----------------	KF030400
*A. longicollis*	HKAS53398	----------------	KF1122376	KF112625	KF112755	KF112238
*A. longicollis*	GDGM42849	----------------	KR052051	KR052058	----------------	----------------
*A. marroninus*	GDGM43288	----------------	NG057040	----------------	KT291753	----------------
*A. miniatoaurantiacus*	HKAS59694	----------------	KT990513	KT990893	KT990352	KT990714
*A. miniatoaurantiacus*	HKAS80485	----------------	----------------	KT990894	KT990353	KT990715
*A. miniatoaurantiacus*	GDGM53350	----------------	MN204532	MN473171	MN549709	MN549678
*A. miniatoaurantiacus*	GDGM43437	----------------	MN204530	MN473149	MN549687	----------------
*A. miniatoaurantiacus*	GDGM43282	----------------	MN204529	MN473148	MN549686	MN549671
*A. miniatoaurantiacus*	GDGM44727	----------------	MN204531	MN473154	MN549691	----------------
*A. mirabilis*	HKAS57776	----------------	KF112360	KF112624	KF112743	KF112229
*A. mirabilis*	REH9765	----------------	KP327661	----------------	----------------	KP327709
*A. moravicus*	MG374a	----------------	KF112421	KF112559	KF112745	KF112232
*A. moravicus*	VDKO1120	----------------	----------------	----------------	MG212615	MG212573
*A. moravicus*	PARMA 154411	----------------	KJ676958	----------------	----------------	KJ676959
*A. nephrosporus*	HKAS67931	----------------	KT990516	KT990895	KT990357	KT990720
*A. nephrosporus*	HKAS74929	----------------	KT990517	KT990896	KT990358	KT990721
*A. projectellus*	MICH KUO 09111014	----------------	MK601708	----------------	MK766271	MK721062
*A. projectellus*	NYBG13392	-------------	KP327622	----------------	----------------	KP327675
*A. projectellus*	AFTOL-ID 713	-------------	AY684158	AY788850	AY787218	----------------
*A. pseudoauriporus*	Farid 501	-------------	----------------	----------------	MW737500	----------------
*A. pseudoauriporus*	JAB 130	-------------	MW662581	----------------	----------------	----------------
*A. pseudoauriporus*	JAB 320	-------------	MW662585	----------------	MW737508	MW737489
*A. quercus-spinosae*	GDGM43755	-------------	KY039967	KY039963	KY039958	----------------
*A. quercus-spinosae*	GDGM43758	-------------	KY039968	KY039964	KY039959	----------------
*A. quercus-spinosae*	GDGM43786	-------------	KY039969	KY039965	KY039960	----------------
*A. raphanaceus*	GDGM44832	-------------	MH670268	MH700218	MH700236	MH700194
*A. raphanaceus*	GDGM52543	-------------	MH670271	----------------	MH700237	----------------
*A. raphanaceus*	GDGM52590	-------------	MH670272	MH700219	MH700238	MH700193
***A. readii***	**Holotype MEXU 30443**	**OR421039**	**OR421566**	**OR49852**	**OR49025**	**OR50124**
***A. readii***	**MEXU 30440**	**OR421040**	**OR421567**	**OR49853**	**OR49026**	**OR50125**
***A. readii***	**MEXU 30439**	**OR421041**	**OR421568**	**OR49854**	**OR49027**	**OR50126**
*A.roxanae*	CFMR BOS 698	----------------	MK601709	----------------	MK766272	MK721063
*A.roxanae*	DS626 07	----------------	KF030311	KF030381	----------------	KF030402
*A. roxanae*	JLF6331	----------------	MH201329	----------------	----------------	----------------
*A. singer*	CFMR BZ 2395	----------------	MK601711	----------------	----------------	----------------
*A. tenuis*	GDGM42601	----------------	KF534789	----------------	KT291754	KT291745
*A. tenuis*	HKAS75104	----------------	KT990518	KT990897	KT990359	KT990722
*A. thibetanus*	HKAS57692	----------------	KT990524	KT990901	KT990365	KT990728
*A. thibetanus*	HKAS76655	----------------	KF112420	KF112626	KF112752	KF112236
*A. thibetanus*	HKAS89494	----------------	KT990525	KT990902	KT990366	KT990729
*A. viscidipes*	HKAS77103	----------------	KT990519	----------------	KT990360	KT990723
*A. yunnanensis*	HKAS75050	----------------	KT990520	KT990898	KT990361	KT990724
*A. zangii*	HKAS74751	----------------	KT990521	KT990899	KT990362	KT990725
*A. zangii*	HKAS74766	----------------	KT990522	KT990900	KT990363	KT990726
*Phylloporus imbricatus*	HKAS 68642	----------------	KF112398	KF112637	KF112786	KF112299
*Xerocomus* aff. *subtomentosus*	HKAS 58865	----------------	KF112389	----------------	KF112630	KF112294

**Table 2:** GenBank accession numbers used for *Chalciporus* phylogenetic analyses in this study. Sequences in bold were generated in this study.

Fungal Taxa	Specimen Voucher	ITS	LSU	*RPB2*	*TEF*
*Chalciporus* aff. *piperatus*	HKAS50214	JQ928610	JQ928621	-------------	-------------
*C. amarellus*	DS46403	-------------	KF303283	-------------	KF030440
*C. citrinoaurantius*	GDGM44480	OM877499	MZ157128	MZ165605	MZ165614
*C. citrinoaurantius*	GDGM44776	OM877502	MZ157131	MZ165608	MZ165617
*C. citrinoaurantius*	GDGM44717	OM877501	MZ157130	MZ165607	MZ165616
*C. citrinoaurantius*	GDGM44481	OM877500	MZ157129	MZ165606	MZ165615
*C. hainanensis*	GDGM44464	OM877505	MZ157127	MZ165604	MZ165612
*C. hainanensis*	GDGM46161	-------------	MZ157126	MZ165609	MZ165613
***C. perezsilvae***	**MEXU-HO 30438**	**OR421044**	**OR421572**	**OR43553**	**OR44012**
***C. piedracanteadensis***	**MEXU-HO 30436**	**OR421042**	**OR421570**	**OR43554**	**OR44013**
***C. piedracanteadensis***	**MEXU-HO 30437**	**OR421043**	**OR421571**	**OR555**	**OR44014**
*C. piperatus*	HKAS84882	-------------	KT990562	KT990397	KT990758
*C. piperatus*	MB04001	OP141440	OP141573	-------------	-------------
*C. pseudorubinellus*	4302	-------------	KF030284	-------------	KF030441
*C. pseudorubinellus*	BN07	-------------	KF030286	-------------	-------------
*C. pseudorubinellus*	DS61207	-------------	KF030287	-------------	KF030441
*C.radiatus*	GDGM43285	KP871804	KF871800	-------------	MZ165610
*C. radiatus*	GDGM50080	KP871806	KP871801	-------------	MZ165611
*C. rubinelloides*	HKAS57362	-------------	KT990563	KT990398	KT990759
*C. rubinelloides*	HKAS74952	-------------	KT990565	KT990400	KT990761
*C. rubinelloides*	HKAS75034	-------------	KT990566	-------------	-------------
*C. rubinelloides*	HKAS58728	-------------	KT990564	KT990399	KT990760
*Chalciporus* sp.	HKAS74779	OP339701	-------------	-------------	-------------
*Chalciporus* sp.	HKAS53400	-------------	OK643702	-------------	-------------

## Data Availability

Not applicable.
